# Supplementary material for: Rapid profiling of transcription factor–cofactor interaction networks reveals principles of epigenetic regulation
Source: Nucleic Acids Res. 2024 Aug 21;52(17):10276–96. doi: 10.1093/nar/gkae706 (PMC11417405; doi:10.1093/nar/gkae706)
Supplement: gkae706_Supplemental_Files [file gkae706_supplemental_files.zip › Supplemental Figure 1-7, Supplmental table 1, & Supplement file legends.pdf]

SUPPLEMENTAL

Supplemental Figures

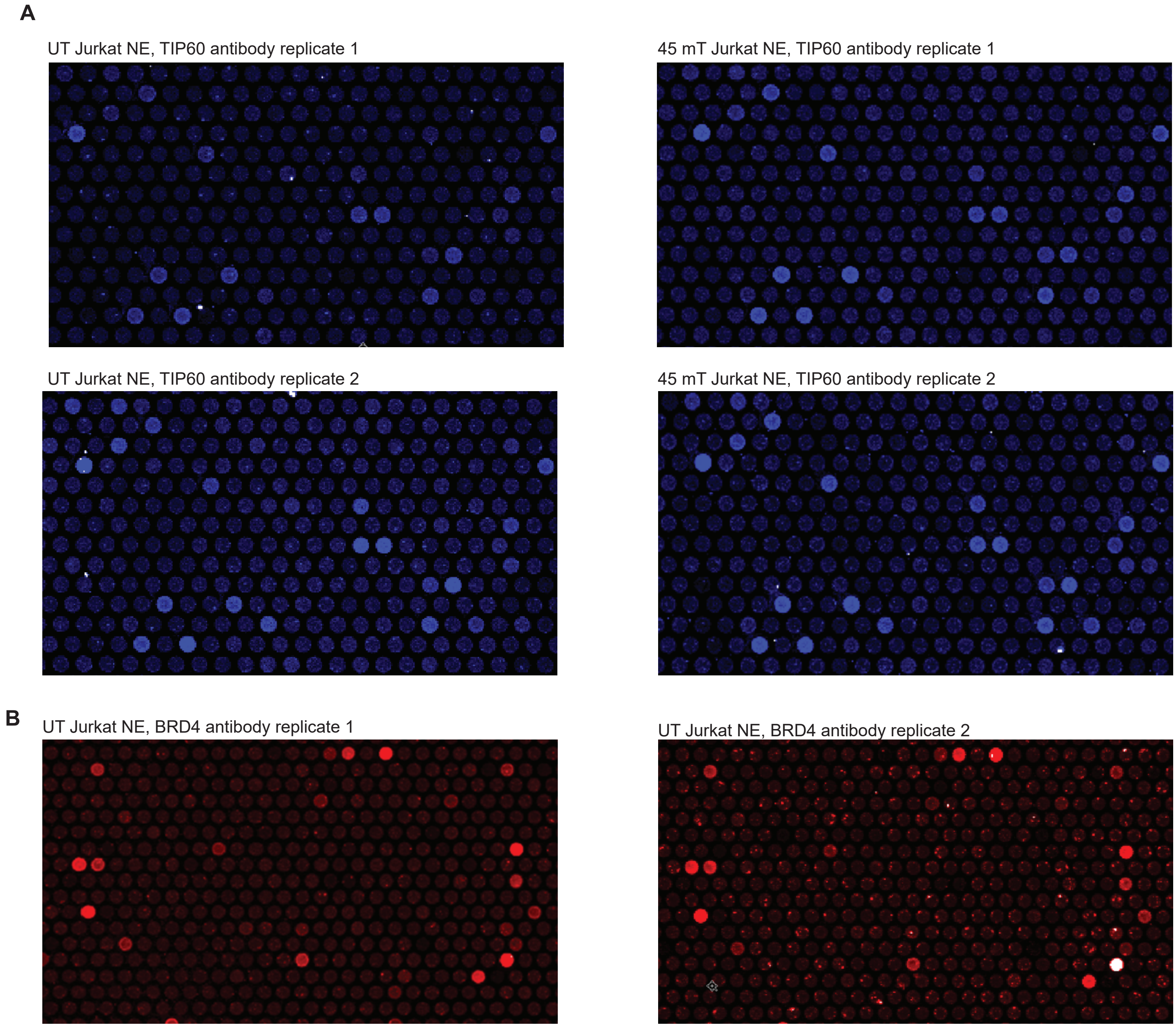

Supplemental Figure 1: Representative scans of microarrays demonstrate reproducibility. The same region of the microarray is shown for two replicates. Fluorescence scans are shown for Alexa Fluor 488 **(A)** and Alexa Fluor 647 **(B)**. Nuclear extracts from untreated (UT) and 45 minutes stimulated (45mT) are shown.

A

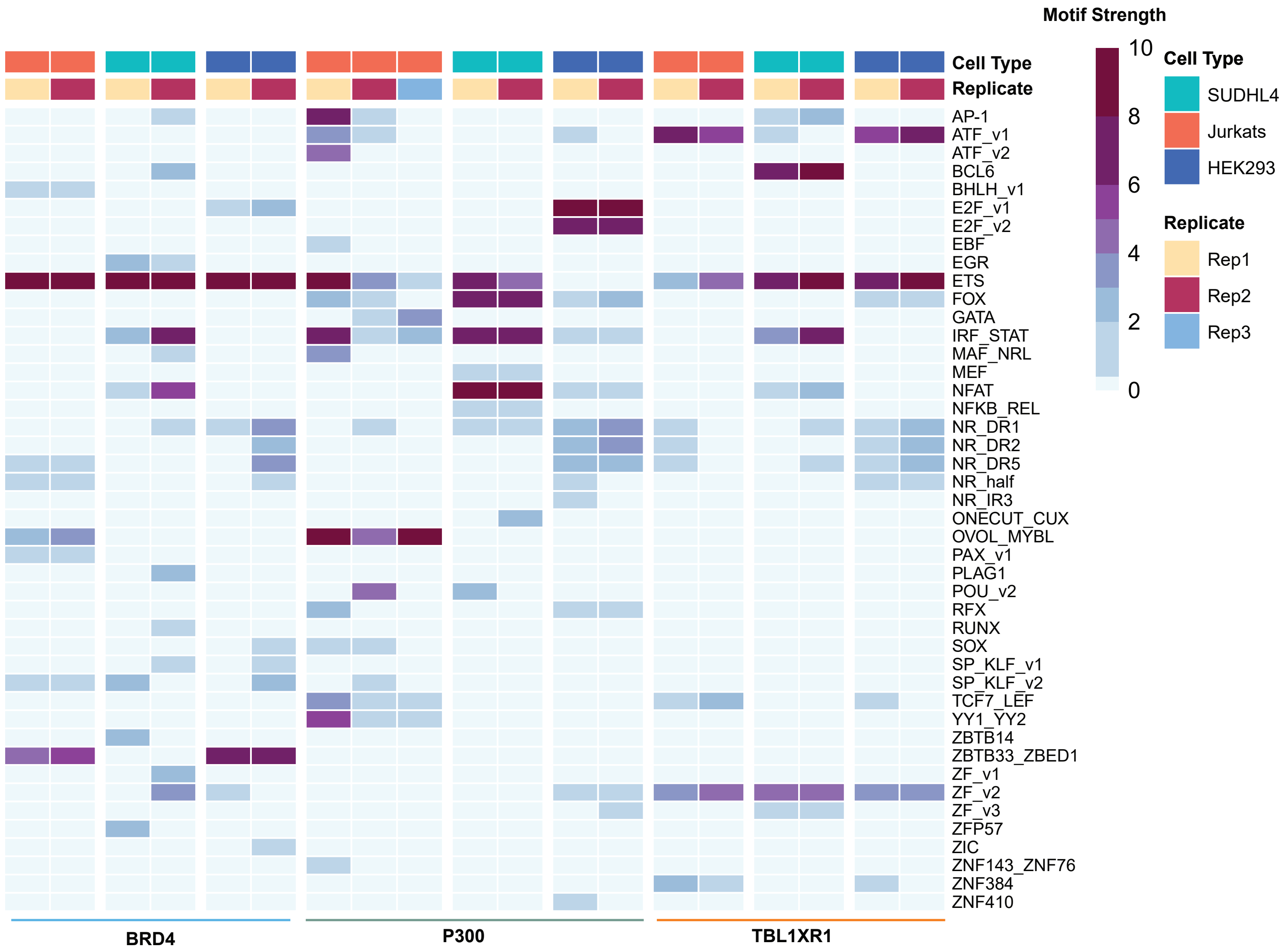

Supplemental Figure 2: CoRec is replicable at TF cluster level. (A) Heatmap indicating CoRec TF-COF interactions colored by motif strength is shown for individual replicate experiments to demonstrate replicability at the TF cluster level.

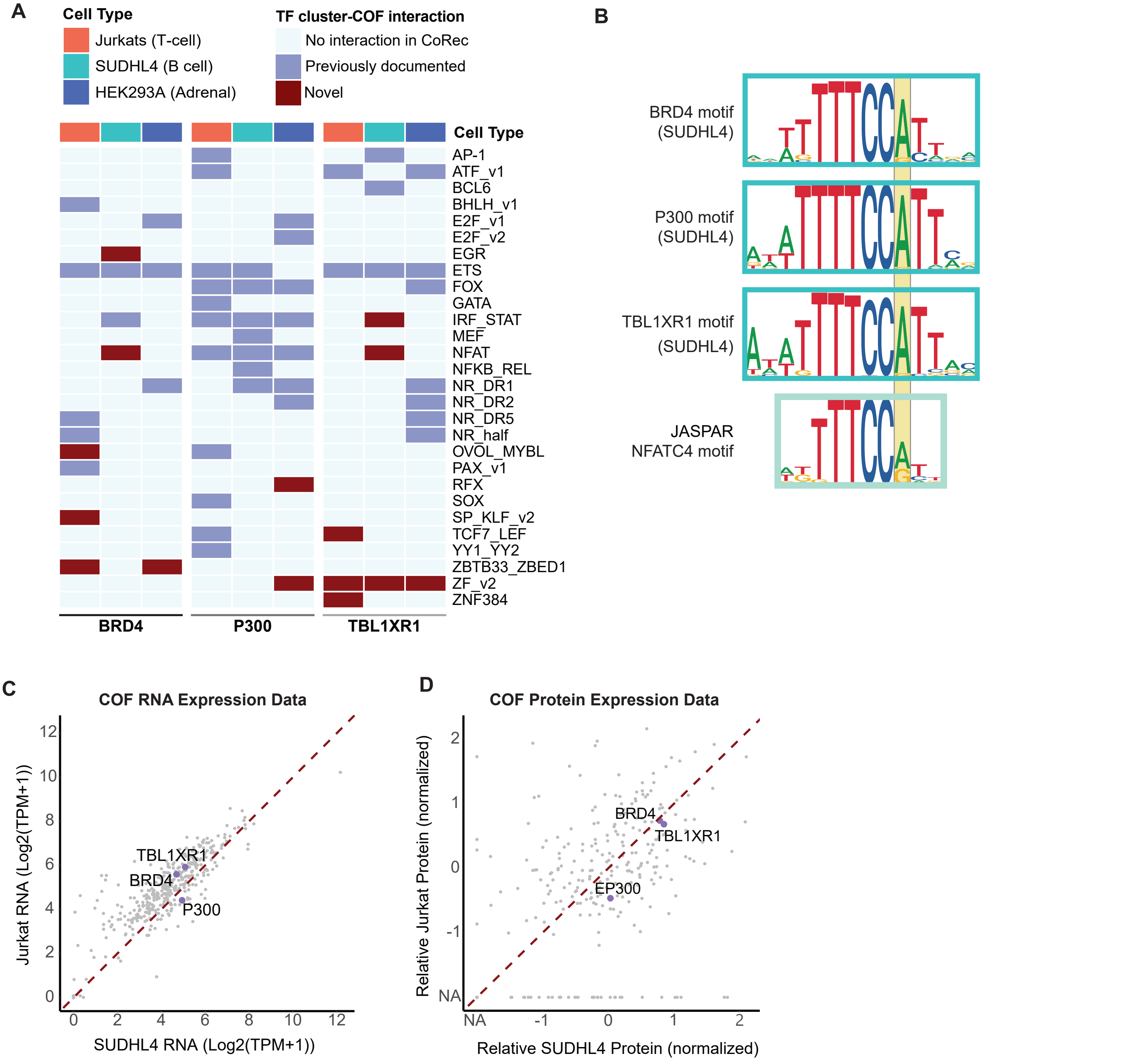

Supplemental Figure 3: Profiling TF-COF interactions provides information on cell-specific TF-COF interactions and TF-COF complex binding motifs. **(A)** Heatmap indicating CoRec TF-COF interactions that have been previously reported in the STRING database. **(B)** COF recruitment motifs in SUDHL4 that match the NFAT cluster and a representative consensus NFAT motif from JASPAR. SUDHL4-specific nucleotide preference highlighted in yellow. **(C-D)** mRNA expression or normalized protein levels for all COFs for unstimulated Jurkat and SUDHL4 with specific COFs profiled highlighted.

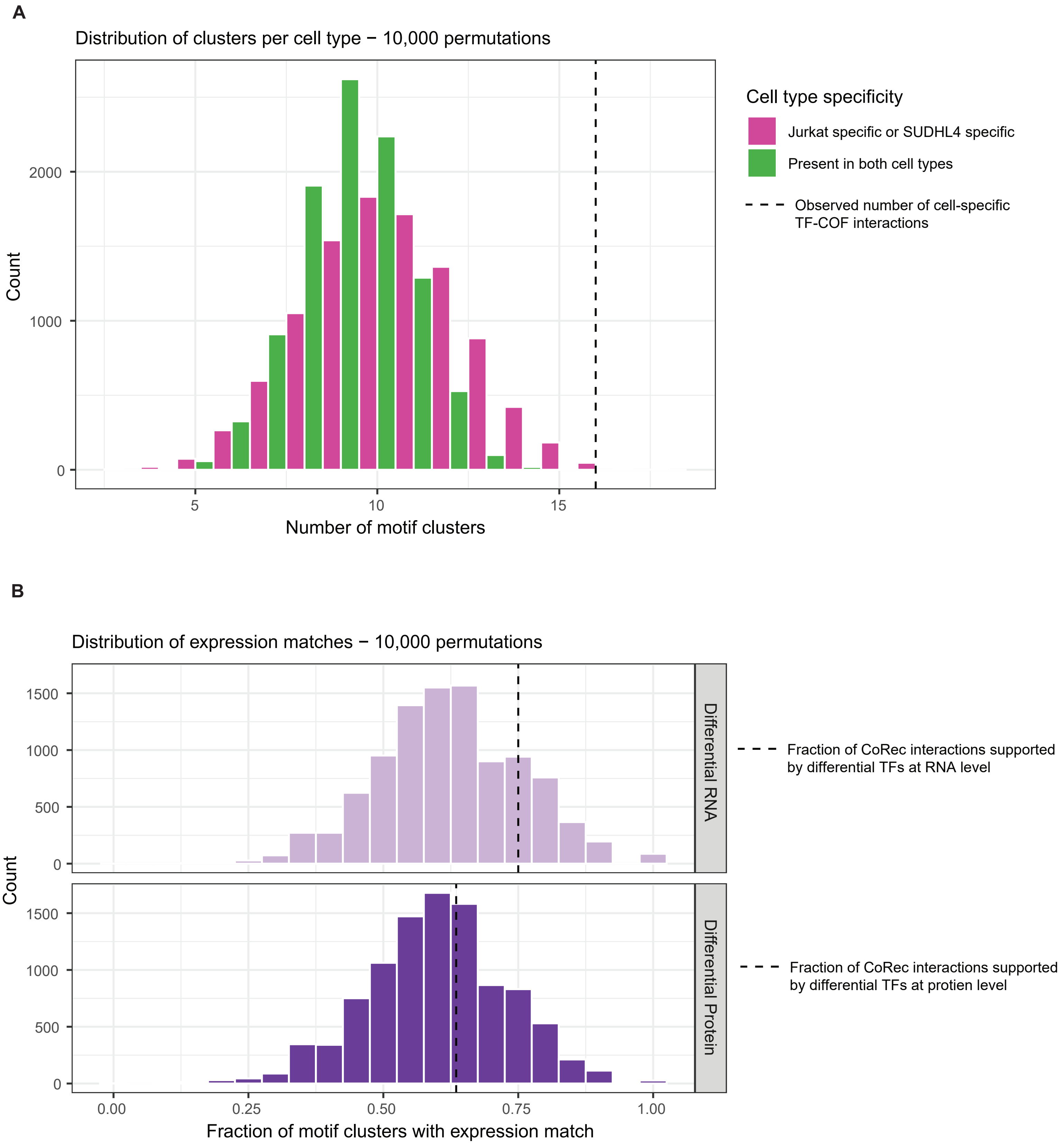

Supplemental Figure 4: Permutation analysis demonstrates significance of cell-specific CoRec observations. (A) Histogram indicating count of cell-specific interactions (pink) or interactions present in both cell types (green) computed over 10,000 permutations of row labels in heatmap from Figure 2A. Permutations were performed independently for each column. The number of observed cell-specific interactions is indicated by the dashed line (2.7 standard deviations above the cell-specific mean). **(B)** Histograms indicating the fraction of cell-specific interactions as determined in (A) that could be explained by differential (log2(FC) > 2) expression (upper) or protein abundance (lower) of at least one TF in the associated TF cluster. The observed number of cell-specific interactions supported by differential expression is 0.28 standard deviations above the mean. The observed number of cell-specific interactions supported by differential protein abundance is 0.94 standard deviations above the mean.

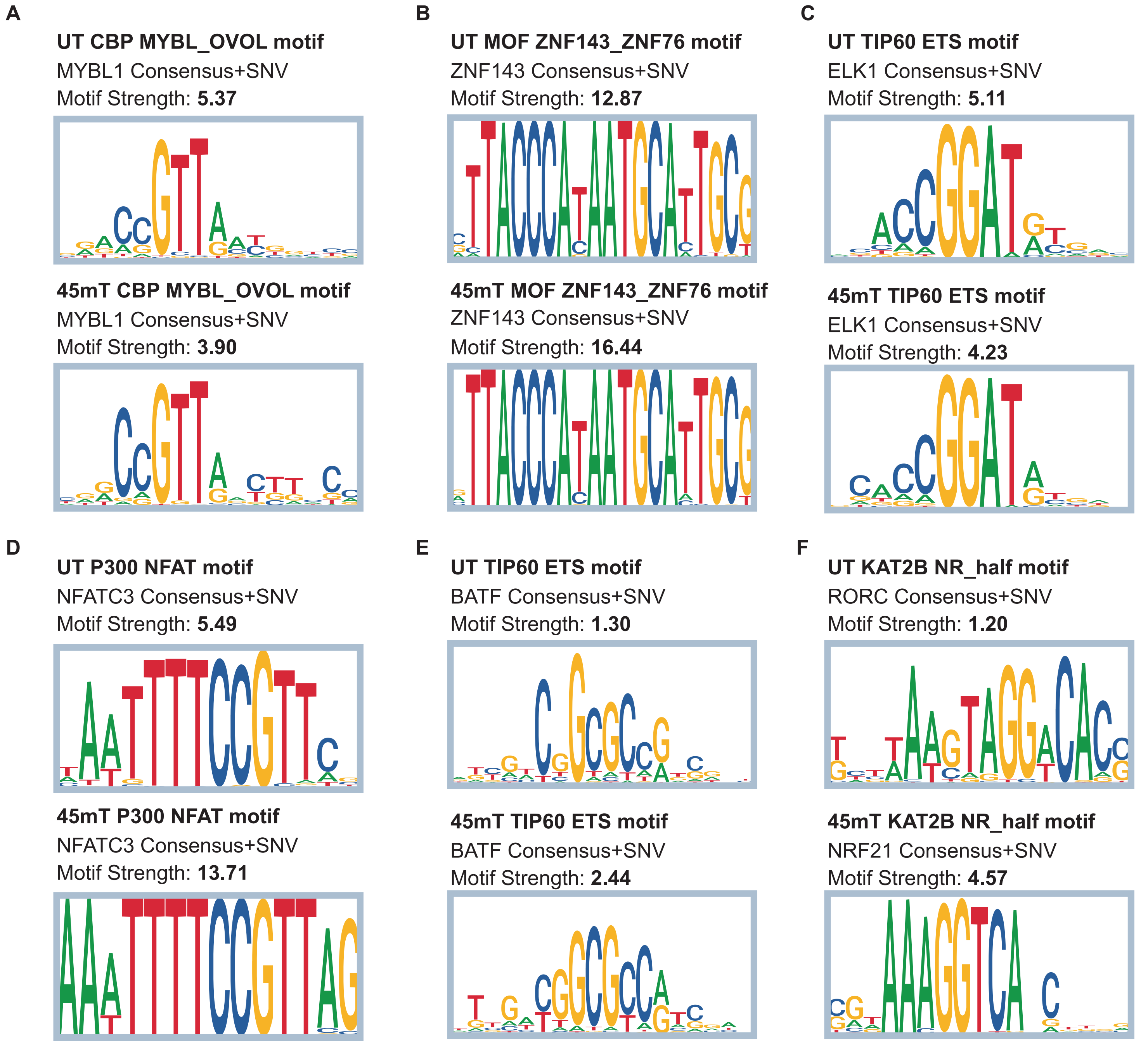

Supplemental Figure 5: Motifs are conserved across motif strengths. (A-F) Representative COF recruitment motif pairs with different motif strengths in untreated (UT) or stimulated (45mT) Jurkat cells are shown. Motifs are annotated with the following information: line 1 – cell condition, COF profiled, and motif cluster match; line 2 – consensus+SV probe set used to define motif; line 3 – motif strength. In (F), motifs are determined using two different consensus+SV probe sets.

A

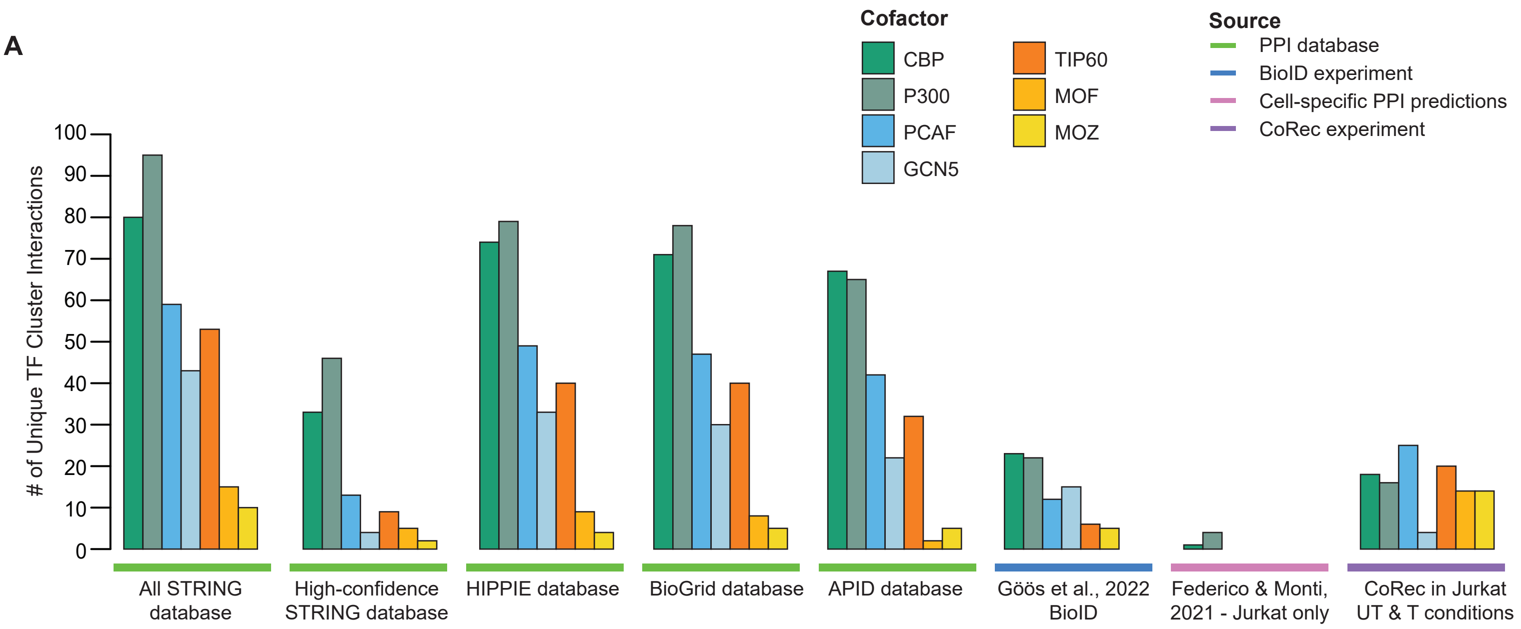

Supplemental Figure 6: Profiling KATs using CoRec produced the expected number of TF-COF interactions. (A) Number of TF clusters identified for KATs profiled in public PPI databases, individual studies, and CoRec experiments.

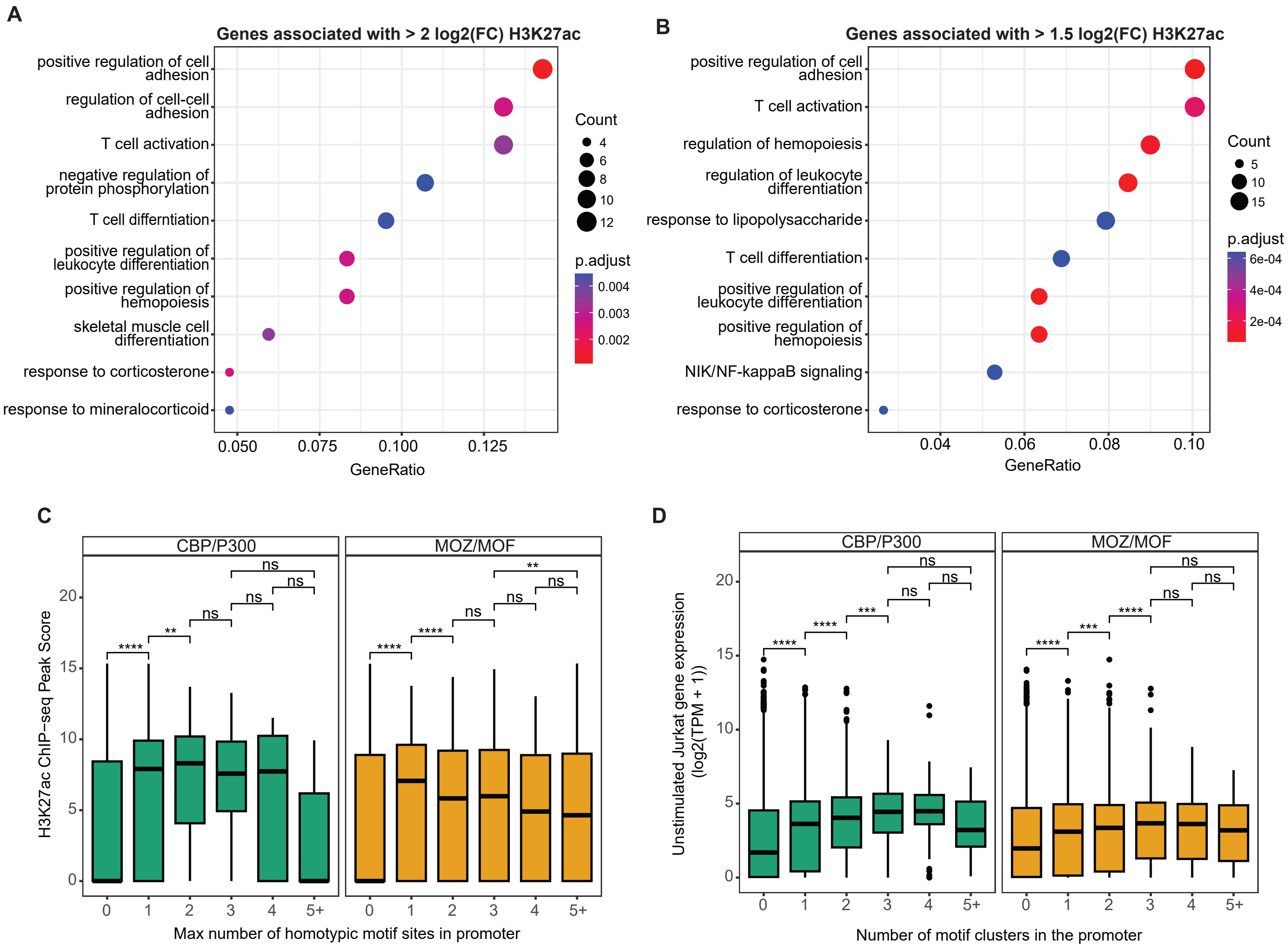

Supplemental Figure 7: Integration of CoRec with other datasets can provide additional insights. (A-B) Top 10 GO enrichment categories for genes associated with stimulus-induced increase in H3K27ac levels. **(C)** Impact of the max number of COF-specific motifs (i.e., homotypic motifs) on promoter H3K27ac levels (peak scores). Motifs evaluated in gene promoters (-500 nt to +100 nt from TSS). Data shown for motifs recruiting CBP/P300 (green) and motifs recruiting MOZ/MOF and not CBP/P300 (orange). **(D)** Impact of the number of COF-recruiting motifs from unique TF clusters (i.e., heterotypic motifs) on gene expression levels. Motif identity and promoter elements defined as in (C).

Supplemental Table

Antibody	Company	Catalog #
**Primary Antibodies**		
CBP	Abcam	ab10489
P300	Abcam	ab14984
KAT2B	Santa Cruz	sc-13124
GCN5	Santa Cruz	sc-365321X
TIP60	Santa Cruz	sc-166323
MOF	Fortis/Bethyl	A300-992A
MOZ	Invitrogen	PA5-66742
TBL1XR1	Santa Cruz	sc-100908
BRD4	Fortis/Bethyl	A301-985A
**Secondary Antibodies**		
Goat anti-mouse IgG (H+L) Highly Cross-Adsorbed Secondary Antibody, Alexa Fluor 488	Invitrogen	A11029
Goat anti-rabbit IgG (H+L) Highly Cross-Adsorbed Secondary Antibody, Alexa Fluor 647	Invitrogen	A32733

Table S1: Antibodies used for CoRec experiments.

Supplemental Files Legends (Files too large to fit into PDF)

Files S1-S2: CoRec-generated z-score motifs.

Files S3-S4: CoRec-generated PPMs.

File S5: TF motifs and assigned clusters.

Files S6-S7: CoRec-generated motifs.

File S8: Differential mRNA expression data for unstimulated Jurkat and SUDHL4 cells.

File S9: Differential protein level data for unstimulated Jurkat and SUDHL4 cells. Files
